# Assessment of NO_2_ Pollution Level during the COVID-19 Lockdown in a Romanian City

**DOI:** 10.3390/ijerph18020544

**Published:** 2021-01-11

**Authors:** Adrian Roșu, Daniel-Eduard Constantin, Mirela Voiculescu, Maxim Arseni, Bogdan Roșu, Alexis Merlaud, Michel Van Roozendael, Puiu Lucian Georgescu

**Affiliations:** 1 Faculty of Sciences and Environment, The European Centre of Excellence for the Environment, “Dunarea de Jos” University of Galati, Domneasca Street, No. 111, 800201 Galati, Romania; adrian.rosu@ugal.ro (A.R.); daniel.constantin@ugal.ro (D.-E.C.); maxim.arseni@ugal.ro (M.A.); bogdan.rosu@ugal.ro (B.R.); lucian.georgescu@ugal.ro (P.L.G.); 2 Royal Belgian Institute for Space Aeronomy (BIRA-IASB), Ringlaan-3-Avenue Circulaire, B-1180 Brussels, Belgium; alexis.merlaud@aeronomie.be (A.M.); michel.vanroozendael@aeronomie.be (M.V.R.)

**Keywords:** COVID-19 lockdown, nitrogen dioxide, in situ, space observations, mobile DOAS observations

## Abstract

This study investigates changes in pollution associated with the lockdown period caused by the COVID-19 pandemic in Galati (45.43° N, 28.03° E), a Romanian city located in the southeast of Romania. The study is focused on nitrogen dioxide (NO_2_), a trace gas which can be related to emissions from industrial activities, heating, and transportation. The investigation is based on in situ observations from local Air Quality Monitoring Stations (AQMS) and mobile remote sensing observations by Differential Optical Absorption Spectroscopy (DOAS) technique. We also show results of the NO_2_ vertical column measured by TROPOMI (TROPOspheric Monitoring Instrument), a space instrument onboard of satellite mission Sentinel-5P, to complement local ground-based measurements. For in situ observations, the lockdown interval (23 March 2020–15 May 2020) was separated from normal periods. The decrease in local NO_2_ concentration during lockdown, measured in situ, is rather small, of about 10–40% at the most, is observed only at some stations, and is better seen during workdays than during weekends. We conclude that the decrease in NO_2_ content over Galati city during lockdown is relatively small and may be attributed to the reduction in local traffic, a consequence of special measures and restrictions imposed during the COVID-19 lockdown by the Romanian authorities.

## 1. Introduction

Coronavirus disease 2019 (COVID-19), caused by Severe Acute Respiratory Syndrome Coronavirus 2 (SARS-CoV-2), is the source of an ongoing global pandemic event [1]. Although not much is known yet, COVID-19 is an acute respiratory disease, which may affect the lungs and respiratory system [2]. The virus spread rapidly at a global level, leading to significant effects on economic losses, besides the effects on human health and mortality [3]. The first Romanian COVID-19 infection was reported on 26 February 2020, when the first five cases of COVID-19 were detected [4]. On 16 March 2020, the first national measures aiming at preventing the spreading of COVID-19 were taken by declaring a state of emergency. One week later, on 23 March 2020, lockdown began, with important restrictions for any travel which was not related to emergency medical care or food supply. The lockdown period ended on 15 May 2020, when the governmental authorities decided to stop travelling restrictions.

Nitrogen dioxide is a trace gas with a high impact on the Earth’s atmosphere. NO_2_ can be released in the atmosphere by natural sources and anthropogenic emissions. The most important sources of NO_2_ are fuel burning, industrial activities, and traffic. During warm seasons and week days, NO_2_ emissions are mostly caused by industrial activities and transportation inside the city [5]. NO_2_ emissions may be higher during cold seasons due to heating, at about 10–40% [6,7]. The severe lockdown, aiming at diminishing infections during the COVID-19 pandemic, presented the opportunity to study NO_2_ variability during a period where some anthropogenic NO_2_ sources were reduced. Some previous studies have shown that the COVID-19 lockdown and the following associated travel restrictions reduced the level of air pollution over many countries which were the subject of a total or partial lockdown [8,9]. NO_2_ atmospheric content varies diurnally and seasonally and from year to year, following changes in meteorological parameters [10], general environmental conditions, and industrial emissions [11]. Assessing whether a decline in NO_2_ is caused by lockdown (via changes in anthropogenic emissions) can be performed if NO_2_ variability during similar periods in other years is investigated. Comparisons with periods before and after lockdown may not be sufficient, since changes in temperature, humidity, or solar radiation that naturally occur may affect the atmospheric NO_2_.

The aim of this work is to assess the effects of lockdown on NO_2_ content in the city of Galati, Romania. This will be performed by comparing NO_2_ content before, during, and after the lockdown in 2020, using TROPOMI, in situ, and Differential Optical Absorption Spectroscopy (DOAS) remote sensing measurements. NO_2_ Vertical Column Densities (VCDs) measured by TROPOMI before, during, and after lockdown are compared with similar periods in 2019. In situ measurements for the first six months of 2020 are compared to their counterparts from 2019. Mobile DOAS measurements performed on 2 April 2020, during the lockdown, were compared to measurements recorded before issuing the lockdown or any other restriction, on 11 February 2020. The study is organized as follows: the data and methodology are described in Section 2, the results and discussions are presented in Section 3, while Section 4 is dedicated to conclusions.

## 2. Data and Methodology

Galati city is located in the southeast of Romania, being in the top 10 Romanian cities by surface and population (~250,000 inhabitants distributed over 246.4 km^2^). The main sources of atmospheric pollution in Galati city are local transportation (traffic) and some industrial activities, which contribute to local enhancements of the atmospheric NO_2_ concentration [12,13,14,15,16].

### 2.1. Instrument Description: Mobile DOAS System

**Figure 1:**
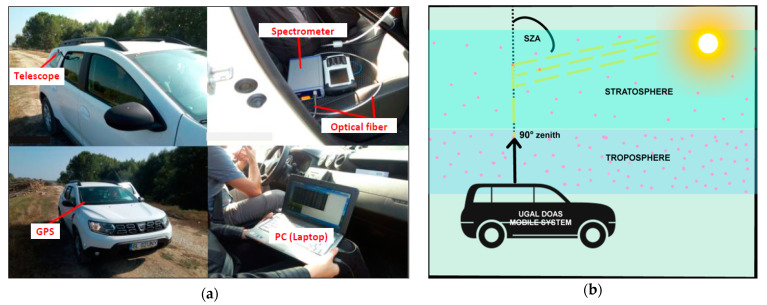
The UGAL mobile differential optical absorption spectroscopy (DOAS) system: (**a**) The components of the UGAL mobile DOAS system; (**b**) Principle of retrieval of NO_2_ amount by mobile DOAS technique [12,13,14,17]; (SZA = Solar Zenith Angle).

The UGAL (University of GALati) mobile DOAS system is a remote sensing instrument used for measurements of trace gases density in the atmosphere. The instrument was developed at the Faculty of Sciences and Environment, University “Dunarea de Jos” from Galati [12,13,14,17] and uses the spectra acquisition software developed at the Royal Belgian Institute for Space Aeronomy (BIRA-IASB). The UGAL mobile DOAS system, shown in Figure 1a, is composed of: a mobile platform (car) and the DOAS system. The main components of a DOAS system are: the optical fiber with a telescope; a GPS mouse used for acquisition of the location of the mobile system; a PC or laptop used for spectrum recording and data analysis. The detection component of the mobile DOAS system is the spectrometer (Avantes ULS2048XL Starline, with a spectral resolution of 0.7 nm and a spectral domain ranging from 295 to 550 nm, manufactured by Avantes The Netherlands). The full specifications of the Avantes ULS2048XL Starline spectrometer and more technical details can be found in [12,13,14,15,17].

The mobile measurements are based on the passive differential optical absorption spectroscopy (DOAS) technique [18]. The DOAS method uses sunlight as a data-gathering channel that integrates the effects of all light absorption caused by particles and molecules encountered on the sunlight paths from the top of the atmosphere to the ground (in this case, the telescope of the system). Figure 1b presents the principle of the mobile DOAS measurement technique. During the mobile DOAS observations, we used only the zenith geometry. The UGAL mobile DOAS system was validated previously in several NO_2_ measurement field campaigns for satellite data validation [5,12,17,19].

### 2.2. DOAS Spectral Analysis and Retrieval of NO_2_ Vertical Column Densities

**Figure 2:**
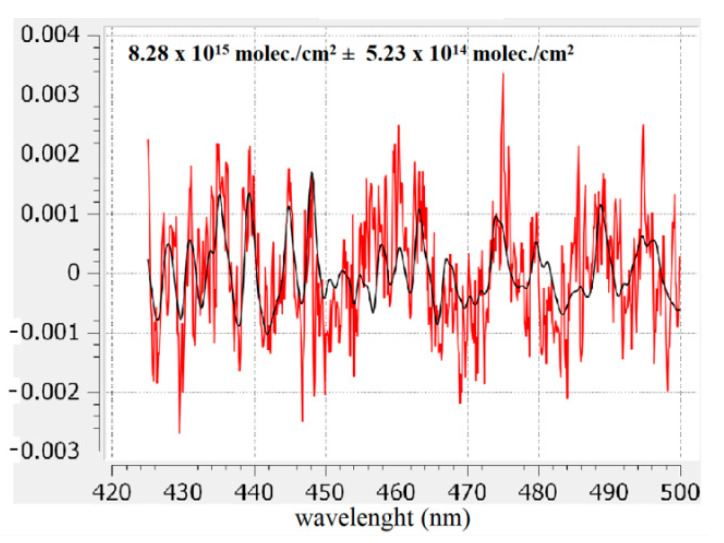
Example of a NO_2_ spectral fitting using QDOAS on a spectrum retrieved on 11 February at 11:28 a.m. in Galati city. The black line corresponds to molecular cross-sections scaled to the detected absorptions in the measured spectrum (red line). (molec. represent molecules).

The recorded spectra using the UGAL mobile DOAS system were analyzed using QDOAS (Qt Differential Optical Absorption Spectroscopy), a specialized software developed at BIRA-IASB (Royal Belgian Institute for Space Aeronomy) [20]. QDOAS is a free, cross-platform, and open-source software that performs DOAS retrievals of trace gases from spectral measurements (satellite, ground-based, mobile, or aircraft-based instruments). This software was validated through many DOAS applications and studies [5,12,13,14,15,16,21]. The result of the spectral analysis is a Differential Slant Column Density (DSCD). The QDOAS settings and the absorption cross-sections used in the spectral analysis for retrieval of NO_2_ DSCD are shown in Table 1. Figure 2 shows the result of a spectral analysis and fitting procedure for NO_2_ DSCD retrieval. Extracting the tropospheric NO_2_ content from DSCDs may require additional information about some physical parameters that describe the atmosphere and radiative transfer conditions (e.g., visibility, albedo, solar zenith angle, etc.) [18,22].

The NO_2_ DSCD retrieved from the spectral analysis in simple terms represents the difference between the NO_2_ slant column density in the measured spectrum (SCDtot) and the NO_2_ slant column density in the reference spectra (SCDref):

DSCD = SCD_tot_ − SCD_ref_ (1)

The SCD_ref_ used in the spectral analysis was recorded using the same mobile DOAS system in zenith geometry on 11 February 2020 around noon (12:24 Local Time (LT)) in a clean and remote area on the E87 road, which links Galati city to Tulcea city, close to Rachelu (45.27° N, 28.37° E). The NO_2_ content in the SCD_ref_ of 4.15 × 10^15^ molec./cm^2^ was obtained by using complementary twilight zenith measurements coupled with the Langley plot method, applied for various Solar Zenith Angles (SZA) intervals as it is presented in [12,14]. Note that we checked the spectral quality of the data by a filtering procedure using O_4_ (collisional complex of (O_2_)_2_) and RMS (Root Mean Square). The O_4_ and RMS are the results of spectral analysis.

The conversion of the NO_2_ slant column density (SCD_tot_) into a NO_2_ Vertical Column Density (VCD) implies another variable which is called Air Mass Factor (AMF). This variable describes the light path throughout the entire atmosphere, and the following relation can approximate it:

(2) $${\mathrm{AMF}}_{\mathrm{tot}}=\frac{{\mathrm{SCD}}_{\mathrm{tot}}}{{\mathrm{VCD}}_{\mathrm{tot}}}$$

where AMT_tot_ represents the total AMF, SCD_tot_—total SCD, and VCD_tot_—total VCD.

Taking into account that the atmosphere is divided into almost distinct layers and most of the air pollutants reside in the lower layers of the atmosphere (i.e., stratosphere and troposphere), Equation (2) therefore becomes:

(3) $${\mathrm{SCD}}_{\mathrm{tot}}{=\mathrm{VCD}}_{\mathrm{tropo}}{\times \mathrm{AMF}}_{\mathrm{tropo}}{+\mathrm{VCD}}_{\mathrm{strato}}{\times \mathrm{AMF}}_{\mathrm{strato}}$$

where VCD_tropo_ represents the tropospheric VCD, AMF_tropo_—tropospheric AMF, VCD_strato_—stratospheric VCD, and AMF_strato_—stratospheric AMF.

Air mass factor, used for the conversion of the measured SCD into VCD, was computed using a Radiative Transport Model (RTM) called UVspec/DISORT (UltraViolet spectral/Discrete Ordinate Radiative Transfer model) [28,29] that was validated through a series of comparisons with other RTMs [30]. The AMF computation requires some parameters that describe the investigated atmosphere: NO_2_ profile, visibility, aerosols profile, albedo, etc. More information about the AMF computation is presented in [14].

The tropospheric column density (VCD_tropo_) can be retrieved through a complex algorithm that is generally described by Equations (4) and (5):

(4) $${\mathrm{VCD}}_{\mathrm{tropo}}=\frac{{\mathrm{DSCD}+\mathrm{SCD}}_{\mathrm{ref}}{-\mathrm{VCD}}_{\mathrm{strato}}{\times \mathrm{AMF}}_{\mathrm{strato}}}{{\mathrm{AMF}}_{\mathrm{tropo}}}$$

For the retrieval of NO_2_ VCD_tropo_ using mobile DOAS measurements performed in Galati city on 11 February (before COVID-19) and 2 April (during COVID-19), we used the following algorithm:

(5) $${\mathrm{VCD}}_{\mathrm{tropo}}=\frac{{\mathrm{DSCD}+\mathrm{SCD}}_{\mathrm{ref}}}{{\mathrm{AMF}}_{\mathrm{tropo}}}-{\mathrm{VCD}}_{\mathrm{stratoOMI}}$$

where VCD_stratoOMI_ represents the NO_2_ vertical column density in the stratosphere extracted from the satellite-borne DOAS ozone monitoring instrument (OMI). The NO_2_ VCD_stratoOMI_ was extracted from the overpass data from OMI above Galati using the Dutch OMI NO_2_ (DOMINO) data product version 2.0 NRT [31] available at [32]. Using this method, we neglected the contribution of stratospheric NO_2_ variation into the extracted troposphere NO_2_.

### 2.3. Instrument Description: Air Quality Monitoring Stations (AQMS)

**Figure 3:**
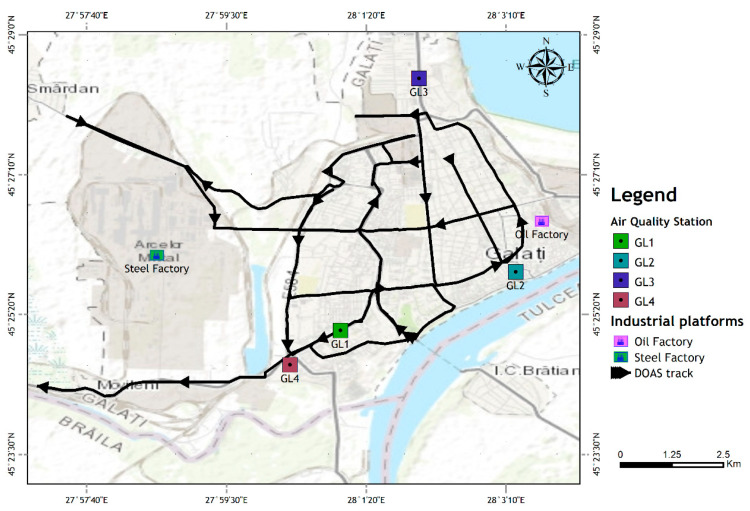
Map of Galati, showing the route of the UGAL mobile DOAS system, the locations of the main industrial platforms (Steel and Oil factories), and of the four Air Quality Monitoring Stations (AQMS) from Galati city (GL1-4).

The air quality assessment network in Galati city is composed of 4 air quality monitoring stations (AQMS), whose location is shown in the map in Figure 3. In general, AQMS are large, acclimatized containers where multiple sensors and equipment are housed for the detection of meteorological and air quality parameters [33,34]. For this study, we used the NO_2_ data retrieved by Ecotech EC9841B (B-Series, manufactured by Ecotech Pty Ltd.) AQMS monitors [35], and wind speed and direction data from an integrated MTX PCTMU000 compact weather station [36], belonging to the local Air Quality Monitoring Network (AQMN). The NO_2_ monitors from the AQMS are based on the chemiluminescence technique to detect NO_2_ [35]. The stations have different detection ranges, which are classified as traffic (code: GL1), urban (GL2), suburban (GL3), and industrial (GL4), and are presented in Table 2. In the following, only the station code will be used (GL1-4). Data used for this study include validated hourly measurements from January, February, March, April, May, and June 2019 and 2020. The in situ NO_2_ data are freely available at http://www.calitateaer.ro/ [33]. NO_2_ loading is expressed in terms of concentration (µg m^−3^). We have used monthly/weekly hourly means, which we calculated as averages of hourly measurements during one month/week.

### 2.4. The Track of the Mobile DOAS and Location of the Local AQMS

The map in Figure 3 shows the traveling route of the UGAL mobile DOAS system on 11 February 2020 (before the lockdown) and on 2 April 2020 (during the lockdown), during the same interval: 09–13 local time (LT). The route was designed to include some of the main streets of Galati city (for traffic emission evaluation), the main local industrial platforms (for industrial emissions evaluation), and passes close to all local AQMS (for a qualitative comparison with the in situ NO_2_). The mobile DOAS measurements were performed in mostly clear sky conditions, as required. The track during the two days was mostly the same, with some minor changes.

## 3. Results and Discussion

The Romanian population was the subject of a total lockdown between 23 March 2020 and 15 May 2020. Movement outside the home or household was prohibited, with some minor exceptions. Schools, malls, shops, and restaurants were closed and most companies started to implement a telework/working from home system.

**Figure 4:**
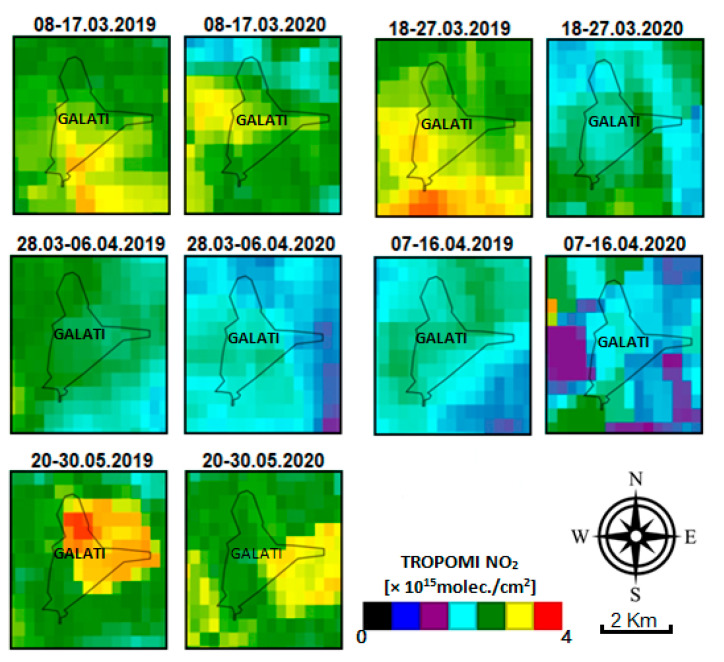
The average NO_2_ VCD over 10 days measured by TROPOMI before (8–17 March), during (18 May–16 April), and after (20–30 May) the COVID-19 lockdown. These are compared with results from the same time intervals in 2019.

Figure 4 shows the spatial distribution of NO_2_ content over Galati city shown by TROPOMI for 2019 and 2020 for intervals corresponding to before, during, and after the lockdown in 2020. To create the NO_2_ maps, we used the offline high-resolution imagery NO_2_ concentrations datasets by Sentinel-5P OFFL NO_2_ (Offline Nitrogen Dioxide, L3), available via the Earth Engine Data Catalog [37]. TROPOMI is a passive imaging spectrometer onboard the S5P satellite platform, covering the UV–visible, NIR, and shortwave IR spectral ranges [38], and measures the atmospheric composition using a pixel size of 7 × 3.5 km^2^ [39]. The tropospheric NO_2_ algorithm uses a retrieval–assimilation modeling system based on the 3D global TM5 chemistry transport model [39]. The TROPOMI NO_2_ data used in this paper are based on the algorithm developments for the DOMINO-2 product and for the EU QA4ECV NO_2_. The resolution of the used data is 0.01 arc degrees. More details about the algorithm calculation of TROPOMI OFFL NO_2_ L3 are presented in [37,38,39].

We used 10-day averages as follows: before COVID-19 (8–17 March 2019/2020), during the COVID-19 lockdown (18 March–16 April 2019/2020), and after the COVID-19 lockdown (20–30 May 2019/2020). The NO_2_ loadings in 2019 and 2020 are relatively similar, on average, during the first interval (8–17 March) and the last one (20–30 May). These correspond to periods before and after the lockdown in 2020. The NO_2_ VCD in 2020 is, in general, slightly smaller than in 2019, but differences are small. Opposingly, the NO_2_ average VCD is clearly smaller in 2020 relative to 2019 for all periods/intervals that correspond to the lockdown in 2020, i.e., 18–27 March, 28 March–6 April, and 7–16 April. However, the reduction level of NO_2_ pollution over Romania, observed by TROPOMI during the partial lockdown, is less prominent compared to the reduction level of NO_2_ pollution observed during the total lockdown of other countries such as China, U.S., Brazil, India, Italy, and Spain [8,40,41,42,43,44,45,46,47,48,49,50,51,52].

The lockdown resulted in a significant reduction in traffic and, partially, in other anthropogenic emissions. We will analyze, in the following, the impact of the lockdown on NO_2_ loading in Galati based on in situ AQMS NO_2_ measurements and remote sensing observations by means of mobile DOAS measurements.

### 3.1. Analysis of In Situ NO_2_ Concentrations for the First Six Months of 2019 versus 2020

**Figure 5:**
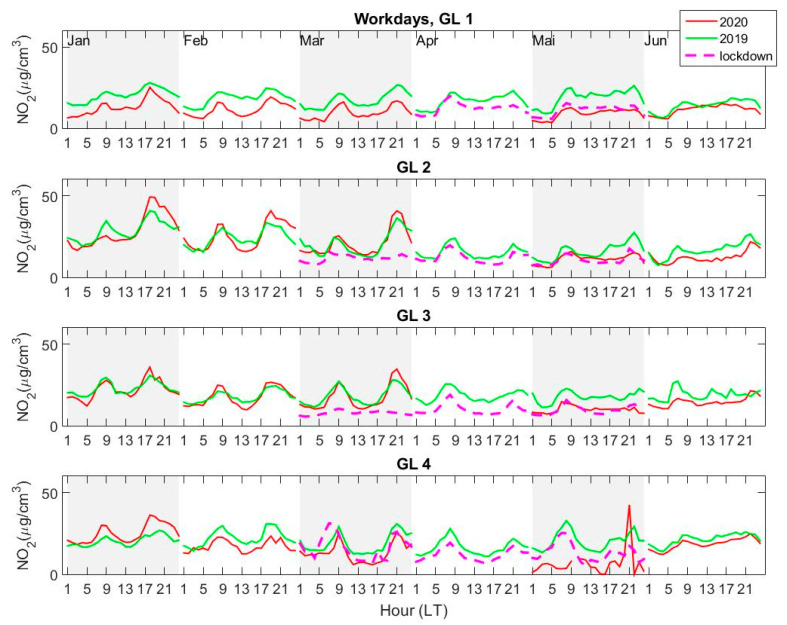
Average diurnal variation of the NO_2_ concentration measured at the four AQMS—GL1, GL2, GL3, GL4—during workdays (Monday–Friday), from January to June 2019 (green) and 2020 (red). The average diurnal variation during the lockdown period in March, April, and May (23 March–15 May 2020) is shown as a magenta dashed line. The diurnal variation during some lockdown periods cannot be seen in some plots because data were not available at that particular station (for instance, March for GL 1).

**Figure 6:**
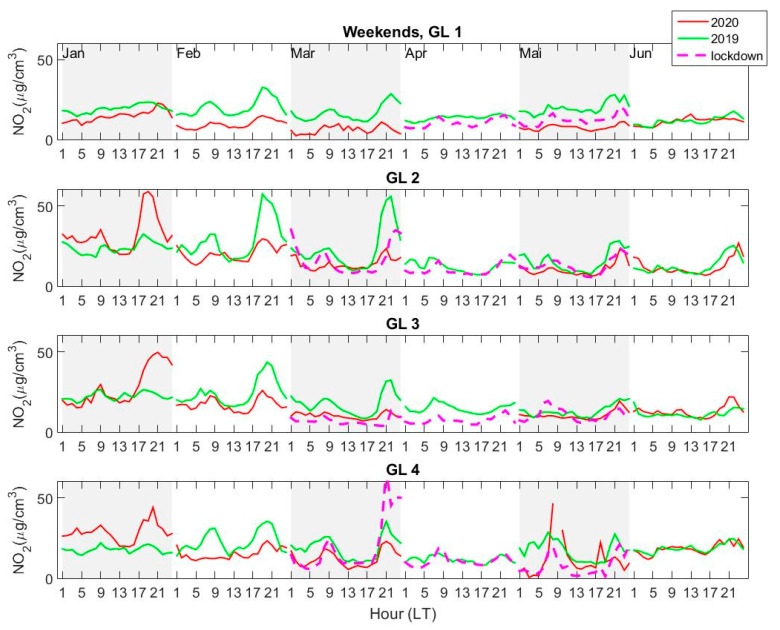
Similar to Figure 5, but for weekends (Saturday, Sunday): 2019—green line; 2020—red line; lockdown (23 March–15 May 2020)—magenta dash line.

The diurnal variation of the average NO_2_ concentrations at each of the four AQ stations was calculated from hourly measurements, for each of the first six months of 2019 and 2020 (January–June). Lockdown weeks, i.e., between 23 March 2020 and 15 May 2020 were considered separately and were disregarded when calculating averages for normal periods in March and May 2020. Additionally, workdays (Monday–Friday) and weekends (Saturday–Sunday) were considered separately. The results are shown in Figure 5 (workdays) and Figure 6 (weekends). The concentration is shown for normal periods in 2020 in order to assess whether the decrease may be considered, at least partially, as a consequence of the lockdown or whether the reduction is a normal feature connected to various other factors, as e.g., meteorological parameters or changes in anthropogenic emissions.

The lowest NO_2_ concentrations are measured, on average, at GL1 and at GL4. Higher concentrations are seen at GL2 and GL3, especially on weekends, which is surprising, since these two stations are considered as urban and suburban, thus should “see” lower NO_2_ concentrations compared to GL1 (traffic) and GL4 (industrial) stations. The well-known diurnal variation with two peaks (morning and late afternoon), previously observed for similar cities [7,10,13,14] and references therein, is seen here as well, especially during workdays, in all months except June (Figure 5). The double peak disappears in June for all stations, in both years. During weekends (Figure 6), the diurnal variation is less regular, with important changes from month to month. Surprisingly, the afternoon–evening NO_2_ average concentrations are sometimes higher during weekends than during workdays at stations GL2 and GL3 for normal periods (January 2020 and February and March 2019). A smaller increase during weekend afternoons is seen also at station GL4 for the same periods. 

The diurnal variation during workdays is more regular at station GL1, and NO_2_ loading is clearly smaller in 2020, in general, compared to 2019, regardless of the time interval (normal and lockdown). There is no difference in the NO_2_ concentration measured at GL1 in 2020 between lockdown and normal periods; moreover, the NO_2_ is lower in May, after the lockdown ended, than during lockdown. Thus, at station GL1, which is in a relatively highly populated area, with normal traffic, there is no real change in NO_2_ loading during the lockdown period. Something similar can be seen at station GL4, where concentrations in 2020 are smaller than in 2019 in all months except January. Here also, the average NO_2_ concentration in May 2020 after the lockdown ended, i.e., after 15 May 2020, is clearly smaller than during lockdown. A peak is seen during the second half of May, close to midnight, whose source is not clear. The daily data show that this peak is indeed seen on 15 May 2020 (which was Friday) in the late evening and may be related to the “release” from the lockdown. The similarity between the NO_2_ variations at the two stations may be explained by their proximity (GL4 is close to GL1 and both are in the Southwest (SW) area of the town). One must also keep in mind that the estimated measurement range of both stations is relatively small (Table 2), which may reduce their sensitivity to local, short-term variations. Thus, stations GL1 and GL4 do not see a significant change in NO_2_ content due to lockdown.

The effect of the lockdown is clearly seen at station GL3 (located on the outskirts of the city, to the North) and, partially, at station GL2, located in a quiet neighborhood, relatively far from industrial sources. During workdays, the average NO_2_ concentration and diurnal variation does not change from 2019 to 2020, except the lockdown period in 2020, when the concentration is clearly smaller compared to the rest of the periods under scrutiny. This does not hold for weekends, when the concentration in February and March 2020 is smaller than in 2019. These two stations have higher measurement ranges, thus they may capture small scale changes in NO_2_ variability. The NO_2_ source at GL3 is mainly local traffic emissions, i.e., people travelling in-between their homes in the surrounding villages, and jobs, inside the city. We infer that the decrease in NO_2_ seen at GL3, especially for March and April, is caused by the significant reduction in local traffic, a consequence of the restrictions imposed on movement outside the home.

A high peak is observed for the weekend during the beginning of the lockdown, in March 2020, in the evening, at station GL4 (and, at a smaller scale, at GL2). Since this is an isolated event, a possible explanation might be related to particular atmospheric conditions with a strong north-eastern wind, carrying NO_2_ from the steel factory towards the city. If this were the case, all stations except GL3 should see this peak, and this is confirmed by the fact that a smaller peak is seen also at GL2. Unfortunately, no data are available at GL1 for that particular time.

If one sticks to the definition of stations from Table 2 (traffic, urban, suburban, or industrial), one may arrive at some false conclusions, i.e., that the observed changes may be attributed to a reduction in a particular type of emissions. However, this is not true, since industrial emissions are carried by the wind; thus, a suburban AQMS from Galati can often detect emissions coming from industrial sources. However, variations in NO_2_ concentration seen on a constant basis may be used for inferring some conclusions about the changes in various anthropogenic emissions, industrial or traffic-related. The variation of the local concentrations and changes of both variations and absolute values point to traffic as the most probable cause of NO_2_ decrease, similar to what has been observed in other cities, such as Milan [8], Madrid [47], or Delhi [9]. However, this reduction is clearly smaller for Galati (roughly 10% or less) mainly because emissions due to traffic in Galati are much lower in general compared, e.g., to very densely populated cities or megacities in Western Europe. Decreases in NO_2_ content of more than 50% [46] were reported for the latter, which were attributed mostly to a mixture of favorable meteorological conditions and traffic reduction and less to industrial emissions changes.

### 3.2. Spatial Comparison of NO_2_ Emissions Measured by AQMS and the UGAL Mobile DOAS System

**Figure 7:**
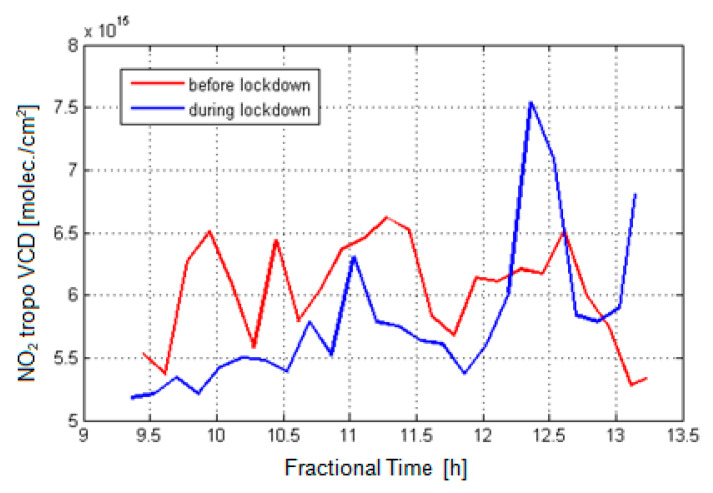
Comparison of the retrieved NO_2_ VCD_tropo_ with NO_2_ differential slant column density (DSCD) for 11 February 2020 and 2 April 2020, derived from mobile DOAS observations over Galati city.

Figure 7 shows NO_2_ VCD_tropo_ results of the retrieval algorithm (described in Section 2.2) for the mobile DOAS observations over two days: 11 February 2020 (before lockdown) and 2 April 2020 (during lockdown). Note that more mobile DOAS observations were not possible during the COVID-19 lockdown due to restrictions.

The NO_2_ VCD_tropo_ observed before lockdown are higher by about 10–20% than the observations performed during the lockdown time interval. The only exception is the measurements performed around 12:20 LT, when the mobile DOAS instrument was crossing the emission plume of a steel factory located nearby Galati city. This may be attributed to the effect of wind and probably to more intense industrial activity.

**Figure 8:**
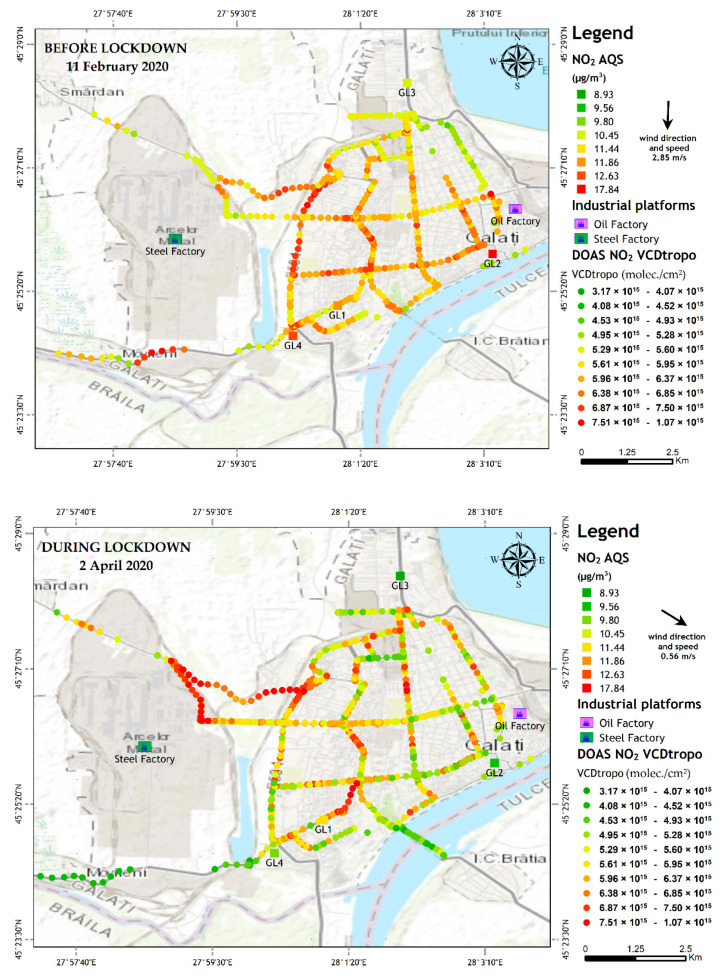
Distribution of NO_2_ VCD_tropo_ measured by the mobile DOAS (colored dots) along the main streets and in situ NO_2_ local concentration (colored squares) at the four stations in Galati on 11 February 2020 (upper map) and 2 April 2020 (lower map). The color of the squares represents the average in situ NO_2_ concentration at the time when the UGAL mobile DOAS system drove by the station.

The spatial distribution of the NO_2_ VCD_tropo_ recorded during 9–13 LT by the UGAL mobile DOAS system on 11 February 2020 and 2 April 2020, respectively, is presented in Figure 8. Figure 8 also shows the in situ concentrations at each AQMS, averaged over the travel time of the mobile DOAS system. The mobile DOAS observations provide a clear picture of emissions propagation and of pollution sources for the two days of measurements.

Both in situ and mobile DOAS observations along the track show that the NO_2_ measured during lockdown is lower than the NO_2_ measured before lockdown. The NO_2_ content, measured by the UGAL mobile DOAS system, is smaller on 2 April 2020 compared to 11 February 2020, along most roads, especially in the center of the town. Hot spots seen in the top plot, mostly associated with crossings, are still evident in the bottom plot; however, the NO_2_ VCD is smaller. The NO_2_ VCD is clearly higher in the western part of the city. The latter unexpected growth of NO_2_ loading can be explained by the northwesterly wind that carries emissions originating at the steel factory towards the city. High values of NO_2_ VCD, between 0.63 and 1.06 × 10^16^ molec./cm^2^, result from steel factory emissions, carried toward the south by the northerly wind on 11 February and towards the western part of the city on 2 April. The spatial distribution of the NO_2_ VCD on 2 April suggests that a NO_2_ emission plume of the steel factory extended over the western and southwestern part of the city, leading to important increases which were also observed near the steel factory by the mobile DOAS but also near GL4.

Table 3 shows the minimum, average, and maximum NO_2_ content observed in situ and from space for the two days. Both in situ concentrations and VCD of the NO_2_ are smaller on 2 April 2020 than on 11 February 2020.

**Figure 9:**
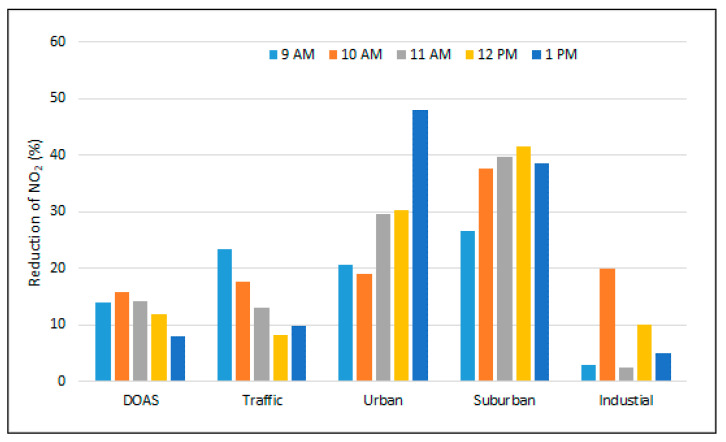
Decrease in NO_2_ content for each hour between 9 and 13 LT in 2 April 2020 (during lockdown), relative to 11 February 2020 (before lockdown), seen in mobile DOAS measurements and in local concentrations measured at the four stations.

Figure 9 shows the relative reduction in NO_2_ content on 2 April 2020 relative to 11 February 2020 for each hour during the experiments, for both DOAS and in situ measurements. DOAS observations are averaged over each hour during the time interval 9–13 LT. The most important reduction in NO_2_ concentration is seen at stations GL2 and GL3, similar to what has been shown in the previous section for most days during lockdown. There is no significant change at GL4 associated with industrial emissions. The lockdown did not shut down all economic activities; most factories continued working, with reduced personnel, but the emissions and industrial process did not stop. Concluding, the reduction in NO_2_ during lockdown compared to normal periods is most likely due to reduced road traffic emissions.

## 4. Conclusions

This study investigates the effect of the lockdown, imposed during 23 March 2020–15 May 2020 to reduce COVID-19 infections, on the NO_2_ pollution level, over an average Romanian city, Galati. This was performed by: (1) comparing the average NO_2_ tropospheric VCD measured by TROPOMI before, during, and after lockdown with similar periods from 2019; (2) comparing in situ measurements of the local NO_2_ concentrations at four AQM stations during the first six months (January to June) in 2020 with similar periods in 2019; and (3) comparing ground-based remote sensing DOAS measurements of the NO_2_ tropospheric VCD on two days—one before lockdown (11 February 2020) and the second during lockdown (2 April 2020).

TROPOMI observations show that the NO_2_ VCD is smaller, on average, during lockdown, compared with similar periods from 2019 but also with periods before and after lockdown in 2020. The local in situ NO_2_ atmospheric concentration is smaller during lockdown only at two out of the four stations, mostly during workdays. Decreases in local NO_2_ concentrations are small (roughly 10%) and are, most likely, due to reduced local traffic. Changes in NO_2_ loading are smaller compared to those observed for larger and more populated cities. Instantaneous changes of up to 40% were observed, but these do not propagate during the entire period or city level.

Ground-based remote sensing measurements were performed using a mobile DOAS system. The NO_2_ VCD observed by DOAS was smaller by ~10% in April, during lockdown, along almost the entire route. Two hotspots were identified in April, which were attributed to industrial pollution carried to the city by the Northwest (NW) wind.

The analyses of both sets of local measurements suggest that the reduction in NO_2_, when observed, is due to the decrease in local traffic. The decrease, in general, is small. Changes in NO_2_ in average cities caused by lockdown do exist, but are less important compared to those observed for heavily populated regions. Additionally, our results show that industrial emissions did not change significantly during lockdown.

## Figures and Tables

**Table 1:** The main settings used for the NO_2_ spectral analysis using QDOAS software.

Absorption Cross-Sections	Temperature	Reference
NO_2_	298 K	[23]
O_3_	293 K	[24]
O_4_	293 K	[25]
Ring	Not available	[26]
H_2_O	296 K	[27]
Polynomial order	5	
Fitting window	425–495 nm	

**Table 2:** Details of the local AQMS from Galati city [34].

Station Type	Station Code	Estimated Range of Detection Maximum (km)	Estimated Range of Detection Minimum (km)	Latitude N (Decimal Degrees)	Longitude E (Decimal Degrees)
Traffic	GL1	0.1	0.01	45.41868	28.016577
Urban	GL2	5	1	45.43146	28.054877
Suburban	GL3	5	1	45.47377	28.033728
Industrial	GL4	1	0.01	45.41117	28.005526

**Table 3:** The NO_2_ detected by AQMS and mobile DOAS (DSCD) on 11 February 2020 and 2 April 2020 during the time interval 09–13 LT.

Date	Measurement Unit	Measured NO_2_			Observations Type
		Minimum	Average	Maximum	
	×10^15^ molec./cm^2^	4.89 ± 0.44	6.04 ± 0.54	10.71 ± 0.96	Mobile DOAS
	µg/m^3^	9.29 ± 0.98	11.86 ± 1.26	13.63 ± 1.44	in situ GL1
11 February 2020	µg/m^3^	11.36 ± 1.20	12.63 ± 1.34	14.52 ± 1.54	in situ GL2
	µg/m^3^	15.15 ± 1.61	17.84 ± 1.95	23.74 ± 2.52	in situ GL3
	µg/m^3^	9.58 ± 1.02	10.45 ± 1.13	12.25 ± 1.30	in situ GL4
	×10^15^ molec./cm^2^	4.40 ± 0.39	5.80 ± 0.52	8.28 ± 0.74	Mobile DOAS
	µg/m^3^	8.52 ± 0.90	9.80 ± 1.05	11.25 ± 1.19	in situ GL1
2 April 2020	µg/m^3^	6.52 ± 0.69	8.93 ± 1.01	11.52 ± 1.22	in situ GL2
	µg/m^3^	9.14 ± 0.97	11.44 ± 1.26	17.43 ± 1.85	in situ GL3
	µg/m^3^	8.02 ± 0.85	9.63 ± 1.03	11.89 ± 1.26	in situ GL4

## Data Availability

Data available on request.
